# Overview of Concrete Performance Made with Waste Rubber Tires: A Step toward Sustainable Concrete

**DOI:** 10.3390/ma15165518

**Published:** 2022-08-11

**Authors:** Jawad Ahmad, Zhiguang Zhou, Ali Majdi, Muwaffaq Alqurashi, Ahmed Farouk Deifalla

**Affiliations:** 1 Department of Disaster Mitigation for Structures, Tongji University, Shanghai 200092, China; 2 Department of Building and Construction Technologies and Engineering, Al-Mustaqbal University College, Hillah 51001, Iraq; 3 Civil Engineering Department, College of Engineering, Taif University, P.O. Box 11099, Taif 21944, Saudi Arabia; 4 Structural Engineering Department, Faculty of Engineering and Technology, Future University in Egypt, New Cairo 11845, Egypt

**Keywords:** waste tires, concrete, aggregate, compressive strength, treatments, durability

## Abstract

Utilizing scrap tire rubber by incorporating it into concrete is a valuable option. Many researchers are interested in using rubber tire waste in concrete. The possible uses of rubber tires in concrete, however, are dispersed and unclear. Therefore, a compressive analysis is necessary to identify the benefits and drawbacks of rubber tires for concrete performance. For examination, the important areas of concrete freshness, durability, and strength properties were considered. Additionally, several treatments and a microstructure investigation were included. Although it has much promise, there are certain obstacles that prevent it from being used as an aggregate in large numbers, such as the rubber’s weak structural strength and poor binding performance with the cement matrix. Rubber, however, exhibits mechanical strength comparable to reference concrete up to 20%. The evaluation also emphasizes the need for new research to advance rubberized concrete for future generations.

## 1. Introduction

**Figure 1:**
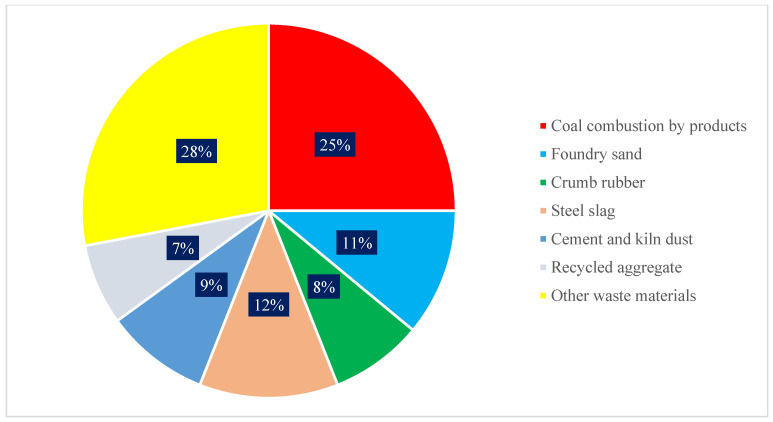
Utilization of different waste materials: data source [6].

Due to the new infrastructure being built, a significant quantity of waste concrete is generated from destroyed buildings every year. Construction and demolition trash is generated annually in China and the EU at 450 million tons and 200 million tons, respectively. About half of the rubber in a tire is made of natural rubber, butadiene rubber, and styrene-butadiene rubber. The other parts include carbon black, metal, textile, zinc oxide, sulfur, and additives [1]. Tires represent environmental dangers owing to their composition, which makes them exceedingly durable, non-biodegradable, and a fire hazard, as well as a breeding ground for rats, mice, and mosquitoes [2]. The proper disposal of used tires has grown to be a significant environmental issue [3]. Each year, the EU member states produce more than three million tons of scrap tires [4], and a stock of 600 hundred tons is present. Due to the desire to create a green environment, keep it clean, and minimize carbon emissions, many western nations, including Canada, are having trouble maintaining and reusing structural waste [5]. Figure 1 shows the recycling percentages of different waste.

**Figure 2:**
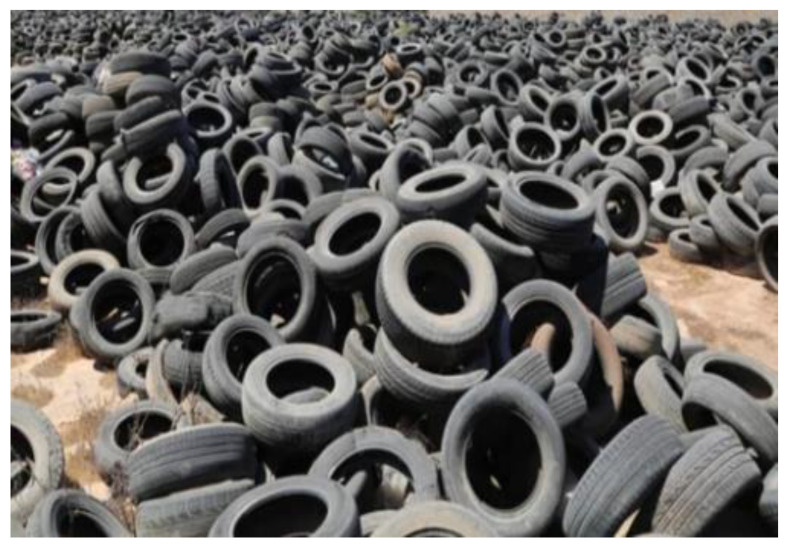
Waste rubber tires [8].

In other words, as seen in Figure 2, these tires from motor vehicles will produce a significant number of waste tires and seriously harm the environment. A total of 290 million tires are produced annually in the United States alone, in addition to the 275 million tires that are already in storage [7]. Therefore, the need for a recycling concept for such used tires has increased.

Recycling used car tires as substitute aggregates to create new-class concrete is a creative solution with favorable effects on the environment, the economy, and performance. Numerous current findings examine the improvement in the compatibility of crumb rubber particles when utilized as a sand substitute. Shredded and/or crumb rubber particles have been extensively explored as a replacement for concrete aggregate [9]. A study concluded that styrene rubber materials in construction buildings improves the quality of concrete for sustainability and durability of the structures [10].

In addition to landfilling, there are other options, such as energy recovery, which is often carried out in cement kilns [11], and pyrolysis of tire rubber to produce carbon black [12]. However, because of the challenges in marketing low-quality pyrolysis end products, the ultimate solution is really not commercially feasible. The recycling of rubber and steel fibers is possible via the process of shredding used tires, which is typically done after an electromagnetic separation.

**Figure 3:**
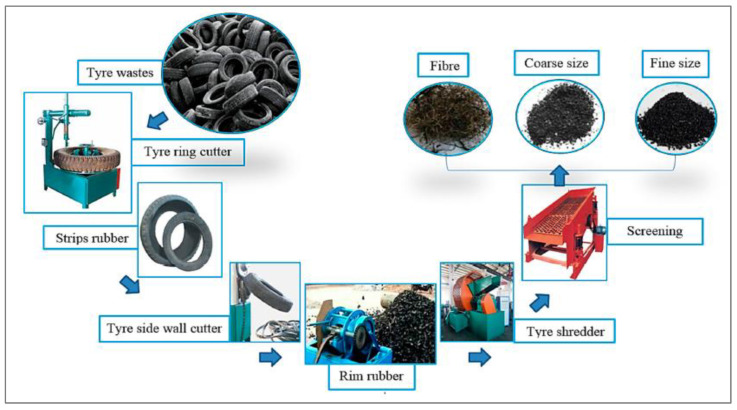
Flow chart of waste rubber tires from waste into concrete [19].

Waste tire features that, if managed improperly, might endanger the environment can be utilized to the building industry’s benefit. Numerous research has been done on concrete that substitutes natural aggregate with scrap rubber from old tires in varying percentages [13]. Figure 3 shows the manufacturing process of waste rubber tires for concrete. The qualities of the concrete produced are dramatically changed when used as a partial replacement for natural coarse and/or fine aggregate. According to studies, adding rubber to concrete regularly lowers the material’s compressive, flexure, and elasticity when compared to normal concrete [14,15]. The weak bond between the rubber surface and paste is primarily caused by the hydrophobic nature of rubber particles and their extreme external irregularity [16]. Based on a microstructural analysis, Taha et al. [17] found that a significant drop in capacity may be related to the behavior of tire rubber particles as a soft aggregate. Concrete’s loss of strength when using rubber as an aggregate has an impact on the rubber’s content, particle size, and characteristics, as well as the mix’s parameters and ratios [18].

Concrete strength qualities are impacted using scrap rubber tires as aggregates. Some of its qualities, including toughness, impact resistance, energy absorption, and sound and temperature isolation, are improved. However, it lessens various other strength characteristics, including workability, split tensile strength, and compressive strength [20]. It is advised to utilize rubber in concrete as foundation pads for rotating equipment and railway stations, as vibration-dampers, or anywhere blast or impact resistance is needed [21]. Additionally, it may be utilized as a shock absorber in structures made to withstand seismic waves [22].

In order to explore sound absorption and the ultrasonic modulus of tire rubber concrete, the research used the ultrasonic echo method to conduct an ultrasonic analysis. The authors came to the conclusion that rubberized concrete is a powerful sound and shaking energy absorber [23]. According to reports, rubber concrete might be utilized to make buildings’ seismic shock-wave absorbers and sound barriers for highway construction [24]. The research looked at the possibility of using scrap rubber from the car sector as fine aggregates in the cementitious matrix to create lightweight building materials. The outcomes of the tests showed that the existence of rubber has a tendency to prevent water propagation and minimize water absorption, providing a superior defense against corrosion for the steel reinforcement [25]. 

Aiello and Leuzzi [26] substituted 0%, 15%, 30%, 50%, and 75% of the sand with the same volume of rubber when adding rubber as a substitute for sand. They observed that because of the reduction in specific gravity, both the density and compressive strength drastically decreased. Atahan and Yücel [27] employed rubber to substitute fine aggregate in a volume ratio of 0%, 20%, 40%, 60%, 80%, and 100%. They observed a steady decline in the samples’ compressive strength until 100% of the fine aggregate was replaced with rubber, at which point 93% of its strength was lost. Additionally, they noted a considerable decrease in the elastic modulus, which reached a 96% loss for 100% replacement of the aggregate. Furthermore, Batayneh et al. [28] replaced the fine aggregate up to 100% by volume with six different mixes including rubber. Their findings demonstrated that rubberized concrete still has enough strength to be employed as lightweight concrete despite the reduction in compressive strength. They advised using these sorts of mixtures for partition walls, traffic barriers, pavement, and walkways in this respect [29].

Brief literature shows that several researchers focus to utilized rubber tire waste in concrete. However, the potential application of rubber tires in concrete is scattered and not clear. No one can easily judge the suitability of waste rubber tires in concrete. Therefore, a compressive review is required to summarize the positive and negative impact of rubber tires as aggregate on concrete performance. The important properties of fresh concrete properties (slump, fresh density, and air content), mechanical strength (compressive, tensile, and flexural strength), and durability (permeability, water absorption, and chloride ions penetration) were considered for analysis. Furthermore, different treatments, microstructure study (scan electronic microscopy), and application of rubberized concrete were also included. Finally, the review also highlights the future research aspects for future generations to further improvement in rubberized concrete.

## 2. Fresh Concrete 

### 2.1. Slump Flow 

**Figure 4:**
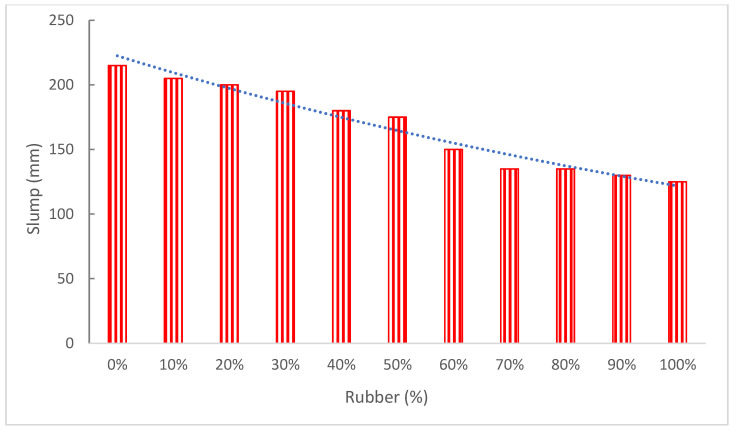
Slump flow: data source [30].

Figure 4 and Table 1 show the flowability of concrete with the replacement of rubber as aggregate in concrete. The flowability of concrete decreased with the addition of rubber as aggregate.

**Figure 5:**
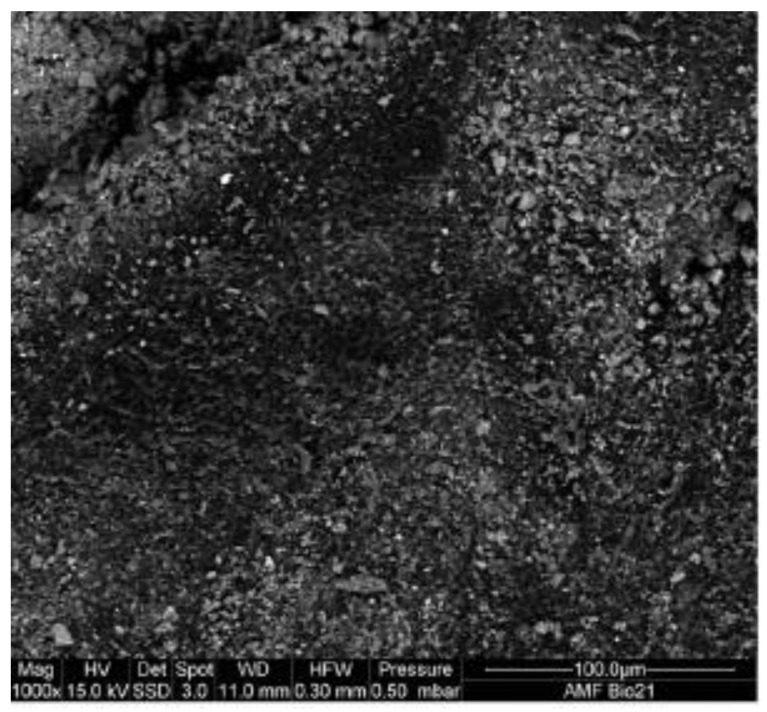
SEM of rubber particle [40]: used as per Elsevier permission.

In addition, mixes created with fine crumb rubber were better to work with than those prepared with coarse tire chips or a combination of tire chips and crumb rubber, according to research. The slump was also shown to diminish as rubber proportion improved [35]. The rubber’s uneven surface roughness caused greater interparticle friction, which led to an increase in the admixture dose [36]. The drop almost remained constant until 12% replacement after having drastically decreased from 140 mm to 110 mm at 24% replacement [37]. The use of rubber aggregates is also believed to reduce slump due to their irregular forms and sharp edges [38]. Similarly, when natural coarse aggregates are partially substituted with rubber aggregates, the slump is reduced as a result of the shape of the rubber aggregate particles [39]. The decrease in flowability of concrete with the incorporation of rubber is mainly due to the rough and angular surface of rubber particles, as shown in Figure 5.

Khatib and Bayomy [41] claim that adding more rubber to concrete reduces both the slump and unit weight. At a 5% crumb rubber content, the slump on rubber without fibers and rubber with fibers fell by 6.67% and 12.50%, respectively. The slump then gradually decreases for rubberized concrete with a crumb rubber content of 10% or 15%. Rubber without fibers and rubber with fibers slumped the least at a 20% crumb rubber concentration, measuring 2 cm and 2.5 cm, respectively. This represents a decrease of 71.43% and 64.29% in comparison to control mixes [42]. 

The fresh mix performance was completely different for materials with a higher natural aggregate to rubber replacement ratio. As additional rubber was included, the droop became worse. However, the nonwetting rubber particles improved the flow of newly mixed concrete [43]. The slump value reduces as recycled concrete aggregate, rubber, and fiber replacement levels rise. The impact of fiber on slump value is greater than that of rubber and recycled concrete aggregate. [44]. The friction force between the components of concrete was enhanced because of the smaller width and longer length. The workability of rubberized concrete demonstrates an increase in a slump as the overall aggregate content rises [32]. Due to their smaller surface area, less paste was needed to coat them. Research, however, asserts that the slump became worse as the rubber dose was increased. The rubber’s nonwetting particles were what made the flow of newly mixed concrete better [45]. Rubber particles may be added to concrete to lower the slump value. As the replacement level increases, hardened rubberized lightweight aggregate concrete loses dry unit weight. This loss of weight causes larger holes in the concrete, which absorb more water and leave no or less free water available for flowability.

Concrete with a larger proportion of rubber particles was less workable. This is mostly because of rubber particles’ greater water absorption rates, which cause the mixture to have less free water overall and, as a consequence, have lower workability. Different sizes of rubber show a decline in flow with a rise in tire particle replacements [40]. 

According to the experimental findings, rubberized concrete absorbed more water than regular concrete, and the amount of rubber in the concrete enhanced the water absorption. Therefore, water, chloride, and chemical assaults on rubberized concrete are more likely to occur [46]. Due to its weak bonding with wet cement paste, the capillary absorption of concrete rises with an increase in the concentration of rubber particles. Since all of the coarse rubber particles have larger absorption rates than the fine rubbers, the size of the replacement aggregate is significant to the increase in water absorption through capillarity [47]. Furthermore, since recycling involves grinding, the surface of the finer waste tire particles is rougher than that of the coarse waste tire. The rubber surfaces of tire crumbs and shreds may be seen in SEM pictures to have rough and jagged surfaces. It is obvious that tire crumbs have more jagged edges than tire shreds [40]. According to research, this roughness increases the frictional resistance to concrete’s flowability, causing mixes with tire crumb replacement to slump less [48]. When compared to concrete without rubber, even though the flow value reduced as the rubber percentage improved, the mixture still provided a workable mix up to a 20% rubber content [42].

### 2.2. Fresh Density and Air Content 

**Figure 6:**
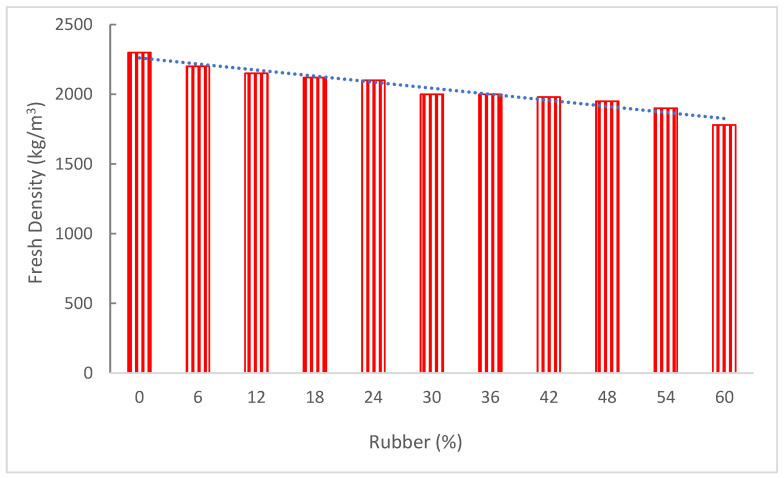
Fresh density of concrete: data source [34].

Air content and fresh concrete density are correlated. Fresh concrete’s density will decline as the amount of air in the mixture rises. Concrete’s properties might alter when different materials are used. It can be noted that the density of various materials differs in the new concrete density test. Figure 6 displays the fresh density of concrete with the incorporation of rubber. It can be noted that rubber decreased the fresh density of concrete. Rubber naturally had a lower specific gravity than the fine aggregate. Because fresh concrete has a low specific gravity, investigations show that its density declined as crumb rubber concentration rose [49]. Research suggested that the density of rubberized concrete improved with the size of the rubber particles, with the lowest density being 1900 kg/m^3^ and the greatest being 2240 kg/m^3^. This is due to the fact that the tiny rubber filled up any gaps between the small concrete particles and served as a fine aggregate, increasing the density [50]. According to research, the spaces around the rubber cause the concrete’s density to decrease as its rubber concentration increases [51]. Gesog Lu [52] found that mixing rubber with concrete resulted in lighter-weight concrete. The densities of the rubberized concrete were 2–11% lighter than those of the reference sample. Due to the increased densification of the concrete structure, Pelisser et al. [53] indicated that although the density of rubberized concrete was 13% less dense than regular concrete, only a 9% loss was seen when silica fume was added to it. Torgal et al. [54] replaced all of the mineral aggregates with crumb rubber and tire chips, replacing plenty of the coarse aggregate. It was noted that the density with the substitute of the sand was decreased by 34%, while the density of the concrete with the replacement of the coarse aggregate was reduced by 45%. The combination resulted in a 33% overall drop in density. 

**Figure 7:**
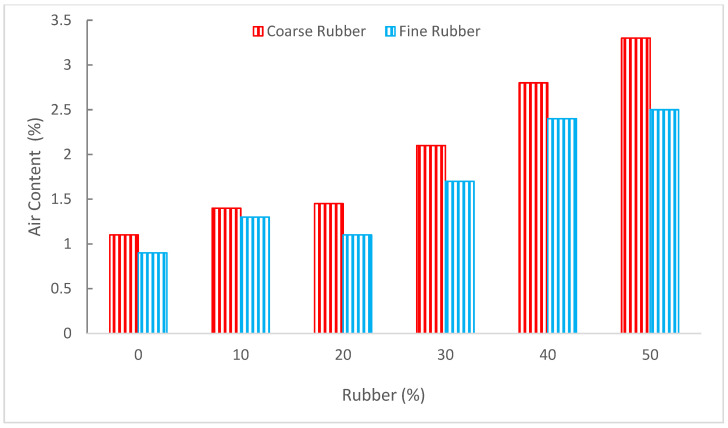
Air content: data source [55].

Figure 7 shows the air content of concrete with the substantiation of rubber as aggregate. The air content improved as the substitution ratio of rubber enhanced. 

However, it can be observed that rubber as coarse aggregate increased air content more than the fine aggregate. When crumb rubber was added to the concrete, Kardos et al. [56] discovered an increase in air content. Due to the large specific area of the fine crumb rubber, as the crumb rubber content grew, the air content of the fresh concrete increased. As a result, increasing crumb rubber (CR) will cause more air to get trapped in the concrete, as indicated by [57]. Due to the rubbers’ non-polar nature, water will be repelled, and air will be readily trapped in the concrete [49]. Richardson et al. [57] compared the air entrainment percentages for conventional concrete, which was 1.9%, and less than 0.5 mm crumb rubber, which was 3.3%. Compared to the sample with less than 0.5 mm crumb rubber, ordinary concrete contained 74% more air than that sample. This is a noteworthy change because a 3% air entrainment is sufficient to provide freeze/thaw protection, especially because the mixture also contains evenly distributed crumb rubber particles that will allow for pressure absorption and have a particle size that is suitable for effective freeze/thaw protection. According to research by Al-Akhras et al. [58], the amount of air in the concrete improves with the size of the tire particle. According to Benazzouk et al. [59], a larger rubber volume ratio results in more air content. The usage of tire rubber ash in concrete may reduce the air content, according to Akhras et al. [60]. With a growth in tire rubber ash content, the mortar’s air content was reduced. In the mortar containing 10% tire rubber ash, the air content dropped from 2.6% in the reference sample to 1.5%.

## 3. Mechanical Strength 

### 3.1. Compressive Strength (CS) 

**Figure 8:**
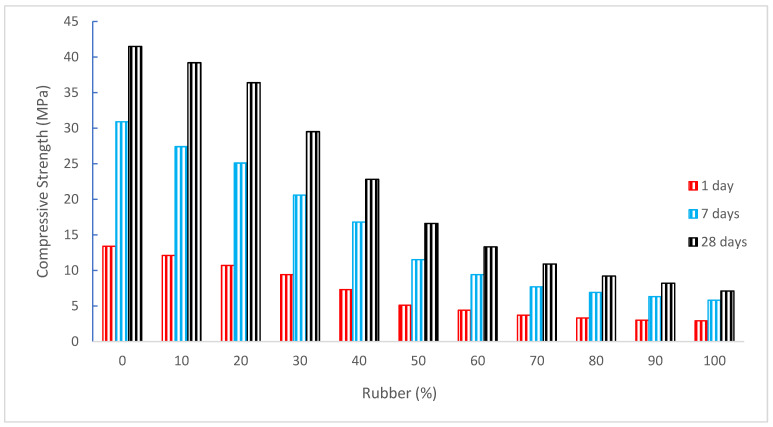
Compressive strength: data source [30].

Figure 8 displays the compressive strength (CS) of concrete with the substitution of rubber as aggregate. It can be noted that CS decreased as the substitution ratio of rubber increased. The qualities of the concrete are dramatically changed when rubber is used as a partial replacement for natural coarse and/or fine aggregate in concrete. According to studies, adding rubber to concrete regularly lowers the material CS and elasticity when compared to normal concrete [14]. Although it is often believed that rubberized concrete has limited mechanical strength, Issa and Salem found that it had high CS [61]. Youssf et al. found that the usage of 10%, 20%, 30%, 40%, and 50% volume rubber/sand substitution decreased the concrete’s CS by 21.3%, 37.9%, 54.3%, 62.5%, and 66.4% at 28 days, respectively, compared to reference concrete [16]. The impact of rubber-based aggregate particle size on rubberized concrete CS. According to the authors, crumb rubber fraction 2/4 lowered CS more than fraction 4/6 [13]. For all samples of recycled rubber mortar, Guelmine et al. [62] noted a rise in the damage factor of both the compressive and flexural capacity, which rose with a rise in the utilized raised temperature.

Depending on the dose of rubber-based aggregate, the strength of concrete containing that aggregate was lowered. The lowest compressive and capacity were seen when rubber fine particles replaced fine natural aggregate by 30% [45]. The scientists also noted that the addition of rubber tire aggregate to concrete results in a reduction in compressive and flexural capacity [63]. Concrete CS is decreased by 10% to 23% when rubber particles are substituted for 7.5% and 10% of the typical concrete aggregate. When rubber is substituted by 7.5% to 10%, the tensile capacity decreases by 30% to 60% [64]. Concrete CS was reduced by 90% when chipped rubber was replaced by 100%. However, when crumb rubber was utilized in lieu of sand in concrete at a 100% replacement rate, the strength was reduced by 80% [65]. When crumb rubber was utilized to substitute fine aggregate, there was a reduction in 28-day CS of 15%, 25%, and 67% for replacement levels of 25%, 50%, 75%, and 100%. Similar to chipped rubber, replacing coarse aggregates with them causes a 40%, 48%, 73%, and 78% drop in 28-day CS for replacement levels of 25%, 50%, 75%, and 100%, respectively [17].

Obinna Onuaguluchi et al. [66] discovered a considerable increase in the combination of coated rubber crumb and silica fume, however, in terms of compressive and tensile capacity. At 5% and 10% sand substitute, this combination performed better than the reference mixture due to the interaction between silica fume and limestone powder. Improvements in the grading of the coarse and fine aggregates may have contributed to a similar small increase in CS of the sample containing 5% chipped rubber [67]. The NaOH-treated rubber particles exhibit improved cement paste cohesiveness, suggesting that the method improved the flexural strength but that the CS decreased by 33% [68]. When tire rubber ash substituted the fine aggregates up to 10%, Smadi [60] noted an improvement in CS. When the tire rubber ash content was 2.5%, 5%, 7.5%, and 10%, respectively, the enhancement in CS of mortar after 90 days was 14%, 21%, 29%, and 45%. According to Feng Lie et al. [69], the CS of rubberized concrete diminishes as the quantity of rubber is enhanced. The CS of rubberized concrete was reduced by larger rubber particles. As a result, rubberized concrete has a greater CS the finer the rubber particles are created.

When 5% of the concrete was replaced, the presence of crumb rubber caused a 63.64% strength reduction relative to the goal strength. CS decreased by 52.73% when 2% of the material was replaced with rubber [70]. Although employing rubber instead of natural aggregates in concrete is a workable solution for recycling scrap rubber tires, earlier research advised against using rubberized concrete for primary structural elements due to its weak CS [71]. According to Meherier [36], concrete CS significantly decreased when rubber content exceeded 20%, although it displayed greater strain capacity compared to control concrete specimens [44]. Comparatively, to control concrete, CS decreases when rubber is added to concrete mixtures; however, it rises as the amount of fiber content increases. The best CS is shown by mix (30% recycled aggregate concrete and 2% fiber content without rubber), which is 26.9% more than the reference sample at 28 days [44]. When coarse aggregates were completely replaced, CS decreased by a maximum of 85%, and when fine aggregates were completely substituted with rubber particles, CS decreased by a maximum of 65% [72]. Due to rubber’s lower elastic modulus compared to natural aggregates and its poor adherence to cement paste, both the CS and elastic modulus decrease. For the finer rubber replacement, the strength loss is less noticeable [40]. The effects of adding waste rubber to composite Portland concrete were explored by Albano et al. [73]. Flowability, density, compressive capacity, and tensile capacity were shown to decline as rubber concentration and particle size rose. The concrete CS and static elastic modulus were all negatively impacted by the use of tire rubber. Higher rubber contents and larger rubber sizes tended to cause a more significant reduction in these strength values [74]. 

The causes of the rubberized concrete’s declining flexural and CS as per past study [67] are, (a) The cement paste, including rubber particles, would encircle the aggregate. Without the rubber, this cement mix would be considerably softer. Due to the fast formation of fractures surrounding the rubber particles during loading, the specimens fail quite quickly. (b) Compared to cement paste and natural aggregate, there would not be a suitable binding between rubber and mortar. As a result of the applied stresses’ uneven distribution, fractures may result. (c) The physical and mechanical characteristics of the component materials affect the CS. Rubber will weaken the materials if it replaces any of them in whole or in part. (d) Rubber has a propensity to rise upward during vibration due to its low specific gravity and lack of adhesion to other materials in concrete. This results in a larger rubber concentration at the top layer. Reduced strengths result from a sample of concrete that is so non-homogeneous. Table 2 shows the summary of CS of rubberized concrete as per past literature.

**Figure 9:**
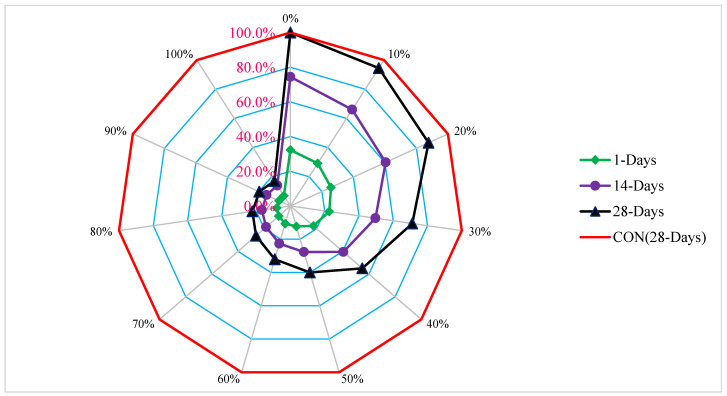
Relative compressive strength.

Figure 9 depicts a relative analysis in which several mixes with increasing percentages of rubber are evaluated using the 28-day CS of the control mix as the reference mix. At a 10% rubber replacement, the concrete strength (CS) is 6% less than the reference concrete’s CS after 28 days of curing.

At a 20% rubber replacement, CS is just 12% less than the benchmark concrete CS. Rubber may be used up to 20% in concrete without having a materially detrimental impact on strength since the CS of concrete with a replacement of rubber is almost equivalent to the CS of reference concrete. However, when the percentages climbed (beyond 20%), a significant decline in CS was seen. Rubber replacement, even at 50%, shows CS 60% lower than reference concrete. Therefore, it is suggested to use rubber up to 20% in concrete. For a higher dose of rubber, treatment of rubber particles should be applied, or it can be used in precast non-load bearing building walls and precast roofs for green buildings. 

### 3.2. Tensile Strength (TS)

**Figure 10:**
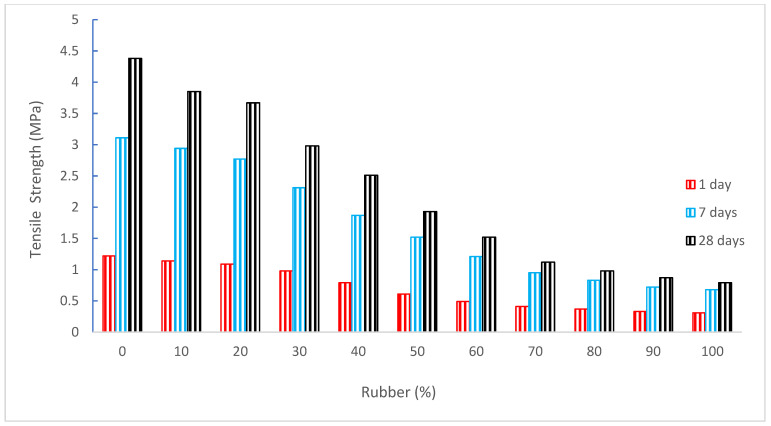
Tensile strength: data source [30].

Figure 10 shows the tensile strength (TS) of concrete with the substitution of rubber as aggregate. It can be noted that TS decreased as the substitution ratio of rubber increased, similar to the CS of concrete. 

Concrete’s compressive strength (CS) is decreased by 10% to 23% when rubber particles are substituted for 7.5% and 10% of the typical concrete’s aggregate. When rubber is substituted by 7.5% to 10%, the tensile strength (TS) of concrete decreases by 30% to 60% [64], which indicate that TS is more adversely affected as compared to CS. Concrete with a 10% replacement of shredded rubber shows a significant drop in TS of up to 76.59%, while concrete with a 5% replacement of shredded rubber shows a reduction of 55.42%. When 1% of shredded tire rubber was added to the overall aggregate composition, a small decrease of 7.62% was seen [70]. Research showed that when the rubber % rose, the values of splitting TS and FS dropped. To obtain results equivalent to the control concrete specimens, the research advised not exceeding a 10% rubber replacement [44]. In the case of rubber, concrete’s splitting tensile strength diminishes as the rubber level rises. Tensile strength (TS) values for 10% recycled concrete aggregate (RCA) are 3.16 MPa, 2.82 MPa, and 2.79 MPa for 0%, 5%, and 10% replacement levels of CR, respectively, when the fiber content is 1%. [44]. Studies have shown that adding fibers to concrete mixtures improves TS and ductility while also lowering the incidence of spalling [82]. As the proportion of rubber substitution in concrete grew, the TS of the concrete decreased. Concrete made using chipped rubber instead of aggregates has a lower TS than concrete made with rubber powder as a cement substitute [83]. However, Farhan et al. [84] found that because of the fragility of these added particles, the TS of rubber is decreased as a result of the addition of rubber particles in cement-stabilized aggregate mixture. However, a rise in post-peak behavior was seen, which led to an improvement in the modified mixes’ toughness. Additionally, the original combinations’ high stiffness was reduced after being partially replaced with rubber particles.

To investigate the strength and toughness characteristics of rubberized concrete mixes, Eldin and Senouci carried out tests. Their findings showed that when the coarse aggregate was completely replaced with chipped tire rubber, the compressive strength decreased by around 85%, while the splitting TS decreased by about 50% [67]. With a higher proportion of rubber substitution in concrete, the TS of the material decreased [85]. Although concrete is a desired and practical material for construction, it has drawbacks, including poor TS, low ductility, low energy absorption, and limited capacity for contraction and shrinkage [30]. In mixes including rubber particles, significant reductions in compressive strength, flexural strength, and breaking TS were observed. When the replacement ratio is 50%, the largest strength loss occurs. It lessens various other mechanical qualities, including slump flow, TS, and CS [11]

Despite losing some of its CS and TS, Deepak and Naidu [86] found that rubber increases the fire resistance of concrete. Due to the rubber aggregate’s poor interfacial connection with the cement paste, the CS and TS of concrete also decrease as rubber aggregate content rise [87]. The findings of the TS test showed that increasing the sand replacement with rubber causes the TS to drop [33]. The results showed that when coarse aggregate was fully replaced by an equal volume of chip rubber, compressive strength decreased by 85%, and TS decreased by 15%, but when fine aggregates were replaced with crumb, CS decreased by up to 65% and TS decreased by 50% [83]. Concrete loses some of its TS when rubber is replaced. TS decreases by around 2–12% when shredded rubber replaces 5–10% of the coarse aggregate [88]. Concrete containing coarse rubber aggregate has a higher strength than concrete containing finer rubber aggregate in the same percentage, as discovered by Mavroulidou and Figueiredo [89]. The control combination yielded the greatest TS and FS, and when the crumb rubber content rose, a systematic decline in strengths was seen [79]. Nearly all replacement levels of treated rubberized concrete are found to have greater FS and TS than standard conventional concretes [90]. According to research, there is an 85% drop in compressive strength and a 50% reduction in TS in rubberized concrete with tire articles and crumb rubber of diameters 36, 24, and 18 mm. However, there is a significant energy absorption [91]. Rubberized concrete’s TS declines, yet the strain at failure rises in line with it [92]. The TS of rubberized concrete that included tire chips and crumb rubber as replacements for aggregates with diameters of 38, 24, and 19 mm was reduced by 50% in tests, but it also absorbed the greatest energy under tensile loading [91]. Table 3 shows the summary of TS of concrete as per past studies.

**Figure 11:**
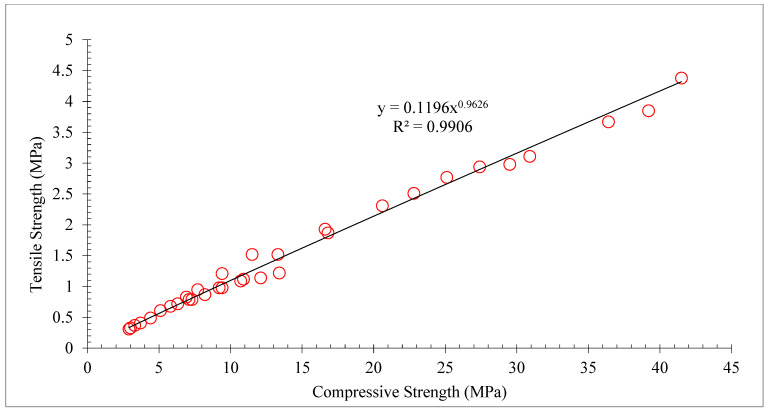
Correlation between compressive and tensile strength: data source [30].

The concrete TS shows the same pattern as the concrete CS. Therefore, a substantial link between the TS and CS of concrete. Figure 11 shows linear regression analysis between CS and TS of concrete. A regression line that has an R^2^ value greater than 90% seems to be straight. Therefore, the equation shown in Figure 11 can be used to predict the TS from the CS of concrete.

### 3.3. Flexural Strength (FS)

**Figure 12:**
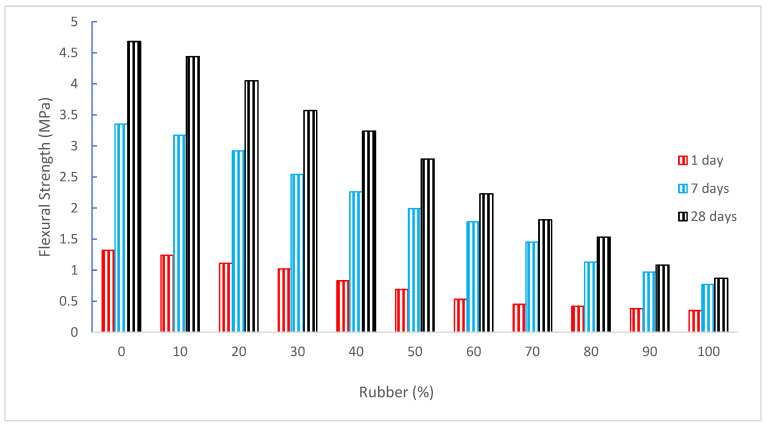
Flexural strength: data source [30].

Figure 12 shows the flexural strength (FS) of concrete with the substitution of rubber as aggregate. It can be noted that CS decreased as the substitution ratio of rubber increased in the same pattern as CS of concrete. According to studies, adding rubber to concrete regularly lowers the material CS, FS, and elasticity when compared to normal concrete [14]. Hora and Reiterman found that elastic modulus and FS decreased very little after 200 cycles of freezing and thawing [93]. With increases in the tire chip contents, FS was found to be significantly less affected than compressive strength. The specimens of FS lost up to 35% of their flexural capacity [63].

FS was found to improve by 12% when shredded rubber was covered with a NaOH solution that was the opposite of what the authors had previously found [70]. A study also discovered that when the crumb rubber (CR) % rose, the values of TS and FS declined. To obtain results equivalent to the control concrete specimens, the research advised not exceeding a 10% CR replacement [48]. FS is seen to rise with rising fiber levels but decreases with increasing recycled concrete aggregate (RCA) and CR levels. The greatest value, which is 8.6% greater than the control concrete mixture, comes from the mix (30% RCA and 2% fiber content without any CR) [44]

The findings of compressive and flexural tests showed that substituting coarse aggregate rather than fine aggregate resulted in a greater loss of mechanical characteristics of rubber concrete. By replacing the coarse aggregate with rubber shreds, the post-cracking behavior of rubberized concrete was favorably impacted, displaying excellent energy absorption and ductility indices in the range seen for fibrous concrete [26]. Concrete’s FS was decreased when rubber was swapped out for gravel or cement. Replacement of coarse aggregates resulted in a decrease of roughly 37%, while replacement of cement resulted in a reduction of 29% [67]. 

The specimens containing tire rubber fibers up to 20% exhibited higher FS than the control specimens [94]. Concrete’s FS decreased for both classes when rubber was substituted for cement or aggregate, although the rate of decline was different [75]. A wider group of studies would enable the optimization of waste tire rubber particle volume percentages and size distribution in concrete to enhance post-cracking behavior while preventing the deterioration of CS and FS [26]. Ganesan et al. [95] confirmed that an increase in crumb rubber % resulted in an increase in the FS of concrete. A small number of investigations, meanwhile, indicated that the FS decreased as the replacement amount of rubber components rose.

In mixes including rubber particles, significant reductions in FS and breaking tensile strength were observed. When the replacement ratio is 50%, the largest strength loss occurs [30]. The investigation of mortars containing TRC led to reductions in FS and a rise in the likelihood of accumulative plastic fractures [96]. The FS decreases as the percentage of rubber used to replace sand increases. For instance, when no finer is employed, a 15% substitution of sand with TRC results in a 17% reduction in FS. However, when fractures emerge, fiber addition may increase FS [33]. The findings showed that although CS decreased by 33%, FS improved as a consequence of this technique. In research, rubber particles were substituted for sand in varying proportions (10% to 100%). The investigation’s findings showed that when the amount of rubber particles increases, mechanical strengths such as CS, TS, and FS decrease. [30]. According to research by Da Silva et al. [97], rubber content in concrete paving blocks ranged from 10% to 50%. The amount of rubber present reduced as the rubber content rose. This decrease for concrete with 50% rubber-containing particles was 32%.

Five indicated rubber contents with volume percentages ranging from 10% to 50% were evaluated by Benazzouk et al. [25]. A rubber volume ratio between 20% and 30% increased the FS despite the fact that the compressive strength was significantly reduced [25]. The results of numerous early studies showed that adding rubber crumbs to concrete improves impact resistance but decreases compressive and FS. There is widespread consensus that the dramatic reduction in mechanical characteristics is brought on by the addition of more rubber [41]. The control combination yielded the greatest TS and FS when the crumb rubber content rose; a systematic decline in strengths was seen [79]. Nearly all replacement levels of treated rubberized concrete are found to have greater FS and TS than standard conventional concretes [90]. Table 4 shows the summary of FS of rubberized concrete as per past literature.

**Figure 13:**
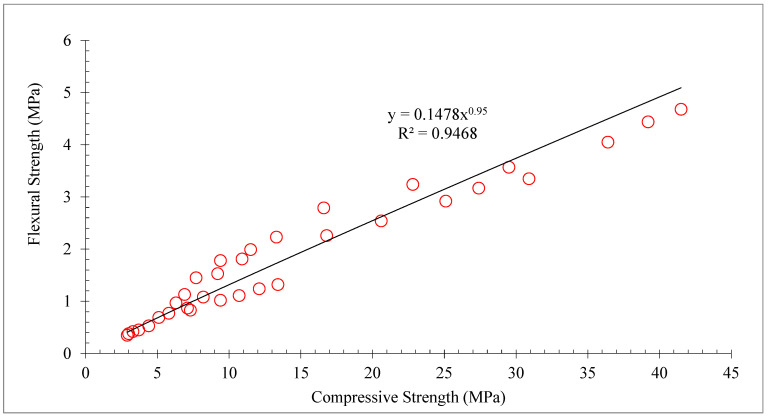
Correlation between compressive and flexure strength: data source [30].

The concrete FS shows the same pattern as the concrete CS. Therefore, a substantial link between the FS and CS of concrete. Figure 13 shows the linear regression analysis between CS and FS of concrete. A regression line that has an R^2^ value greater than 90% seems to be straight. Therefore, the equation shown in Figure 13 can be used to predict the FS from the CS of concrete.

### 3.4. Failure Modes 

**Figure 14:**
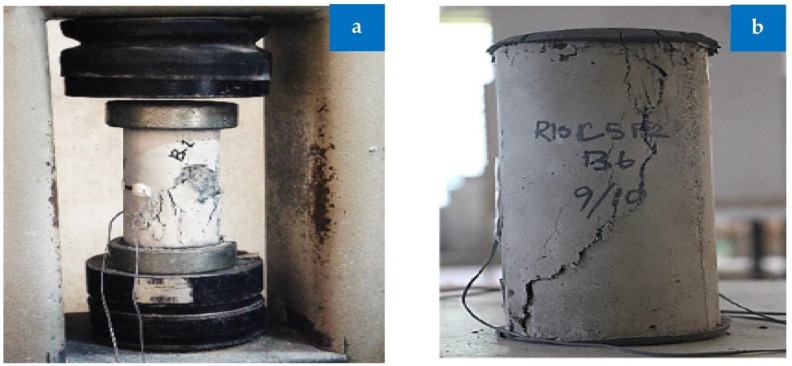
Cylinder failure under compression: (**a**) reference and (**b**) combination of RCA, rubber, and fibers [44]: used as per Elsevier permissions.

The concrete cylinder’s failure pattern during a compressive strength test is seen in Figure 14. Most of the concrete cylinder’s failures were of the cone and shear types. Contrary to the control batch’s brittle collapse, the concrete cylinders containing rubber and fiber showed a gradual and progressive failure. In contrast to sand, rubber is softer than that material, making it more pliable under compression. Fiber is effective in preventing cracking because it creates a strong bridging effect between aggregate and cement paste and maintains the aggregate particle’s cohesiveness.

**Figure 15:**
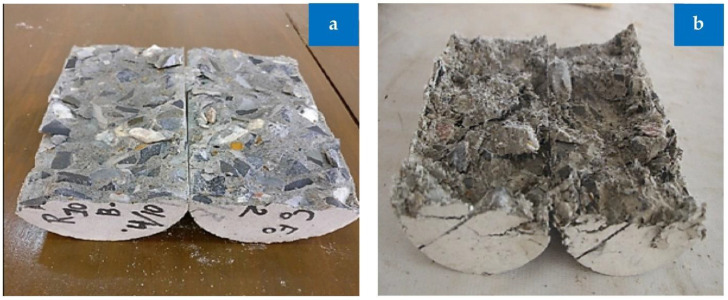
Splitting cylinder failure: (**a**) reference and (**b**) combination of RCA, rubber, and fibers [44]: used as per Elsevier permissions.

The rubberized fiber-based concrete cylinders exhibited uneven failure planes because the fiber keeps the aggregate and cement paste together to prevent the fracture, but the control mix and the mixes with simply RCA replacement broke along well-defined failure planes, as seen in Figure 15. 

**Figure 16:**
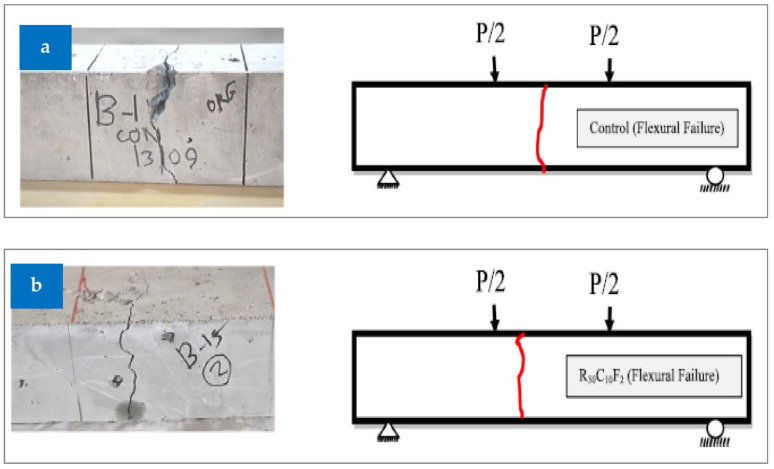
Beam failure: (**a**) reference and (**b**) combination of RCA, rubber, and fibers [44]: used as per Elsevier permissions.

The failure pattern of the concrete beam during an FS test is seen in Figure 16. In the control and just recycled concrete aggregate (RCA) replacement specimens, the load-deflection curve quickly plummeted and split into two halves. When the maximum stress was applied, the beams with fiber, especially the combinations with 2% fiber, did not completely fracture as they did with other specimens. As a result of their more ductile behavior, the collapse occurred gradually. In summary, fiber-containing concrete exhibits higher hardness ratings and is more ductile when compared to control concrete mixtures. Later, as shown in Figure 16, those beams are physically cracked to investigate the failure surfaces. On each side of the crack near the point of collapse, it is evident that the fiber builds a bridge within the concrete matrix.

## 4. Enhancing Properties of Rubber

### 4.1. Water Washing

The rubber is cleaned with water to remove additives, organics, contaminants, and dirt that were placed on its surface during manufacture. According to certain studies, rubberized concrete made with rubber that has been cleaned with water is a little bit stronger than the rubberized concrete used as a control. The cement’s compressive strength rose by 15% [98]. When compared to a 24 h soak, a 2 h soak of crumb rubber in tap water yields far superior results for the rubber mortar’s strength and bonding abilities. In addition to enhancing the interfacial transition zone between the rubber particles and the cement paste, it also exhibits a higher compressive strength after 28 days when compared to crumb rubber mortar that had been soaked for 24 h [99].

The strength of rubberized cement cannot be increased only by water washing. Water cleaning, on the other hand, may effectively remove rubber contaminants that are soluble in water before adding the combination since it is the most cost-effective and ecologically beneficial way. It shows a positive development in the rubber exterior’s hydrophilicity [100]. There is little information accessible in this area.

### 4.2. Silane Coating Agents (SCA)

**Figure 17:**
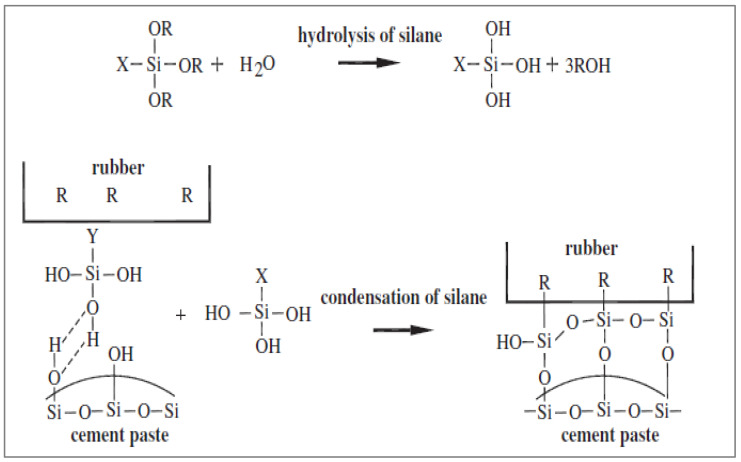
Mechanism of silane coating agents [105]: used as per Elsevier permissions.

The only chemically altered rubber surface is the latex treatment. This procedure involves soaking rubber in a solution containing CS2, KMnO4, acid, SCA, acetone, Ca(OH)_2_, and NaOH, followed by UV exposure and partial oxidation. Chemical treatment cleans the oil from the rubber surface, gets rid of the dust and grime, and makes the rubber more hydrophilic and uneven. By modifying the rubber with NaOH liquor, rubberized cement characteristics are most often improved. This is due to the improved wear resistance, fracture energy, and FS shown by rubber that has been treated with NaOH liquid. In addition, the research found [53] that the combination of silica fume (SF) and NaOH treatment might produce an interfacial transition zone (ITZ) that is more stable. The enhanced water affinity of rubber following the surface treatment may have contributed to the small rise in the density of rubber-modified cement composites after the silane coupling agent was applied to the rubber particles’ surfaces. Another explanation might be that the air gaps surrounding the rubber particles decreased as a consequence of the chemical link that formed between the rubber particles and cement hydration products. The increase in density for cement composites incorporating silane-treated rubber varied from 1.8% to 3.6% for the 5–25% rubber concentrations employed in the research. The improvement in compressive strength of rubber-modified cement composites was more significantly impacted by the silane coupling agent treatment than was the increase in density. The compressive strength of cement composites made with silane-treated rubber was 24%, 9%, 18%, 14%, and 22% higher than that of the paste containing as-received rubber, respectively, at rubber contents of 5%, 10%, 15%, 20%, and 25%. The chemical link created by the silane coupling agent between rubber particles and cement paste was primarily responsible for the increase in compressive strength. Figure 17 depicts the silane coupling agent’s reaction process. Methoxy groups and reactive vinyl or epoxy group (X) are found in silane coupling agents (OR). The methoxy group becomes the hydroxyl group by hydrolysis (OH). The OH groups are further chemically or physically attached to an inorganic substance via dehydration condensation or a hydrogen bond (cement paste). An organic substance is chemically joined to the X group (rubber). The chemical bonds created by the silane coupling agent will need more energy to break, resulting in enhanced compressive strength of the composites containing silane-treated rubber.

According to research [101], treating rubber with NaOH liquid reduces the mixture’s plummet by 25%. Additionally, NaOH treatment may help make rubberized cement more durable. A reduction in the mixture’s resistivity and an increase in the adherence of the rubber to the cement were also effects of the alteration of the NaOH solution [102]. The specimens with reduced electrical resistance have enhanced long-term durability. By promoting adhesion at the interface, the SCA functions as an aggregate to improve adhesion between the concrete matrix and rubber. It causes the cement matrix and rubber to physically and chemically mix to form a firmly linked structure by acting on the inorganic/organic interface section. According to research [103], the rubber’s tensile stiffness and strength are enhanced after SCA processing. According to a study [104], the hydrolysate of SCA may react with concrete paste to strengthen their bond and noticeably improve the microstructure. The SCA treatment, which results in the FS and CS of rubberized mortar, may significantly enhance the mechanical performance of the rubberized cement. When rubber was treated by Guo et al. [102] utilizing SCAs, the bond between the rubber aggregates and the cement paste was enhanced.

### 4.3. Cement Coating 

The density and compressive strength of rubber-modified cement composites were further improved by a cement coating created using a silane coupling agent around rubber particles. The 30% cement coating was more successful in improving density than the 60% cement coating. The compressive capacity of concrete modified with rubber was greatly enhanced when the silane coupling agent was combined with cement coating as opposed to the silane coupling agent alone. At the rubber contents utilized in the investigation, the silane coupling agent surface treatment increased compressive strength by 9% to 24%, but the 30 weight % cement coating increased compressive strength by 27% to 110%. The increase in compressive strength was significantly greater for the 60 weight % cement coatings, ranging from 53% to 168%. The rubber-modified cement composite was able to preserve a considerably greater percentage of the compressive capacity of the reference pastes without rubber, thanks to the rubber’s considerable rise in the compressive capacity as a result of its two-staged surface treatment. For instance, the composite comprising 60% cement-coated rubber could sustain 94% of the strength of the reference paste at a rubber level of 5%. The compressive capacity formed with 60 weight % cement-coated rubber was greater than 50% from the control paste, even at a rubber content of 15%.

**Figure 18:**
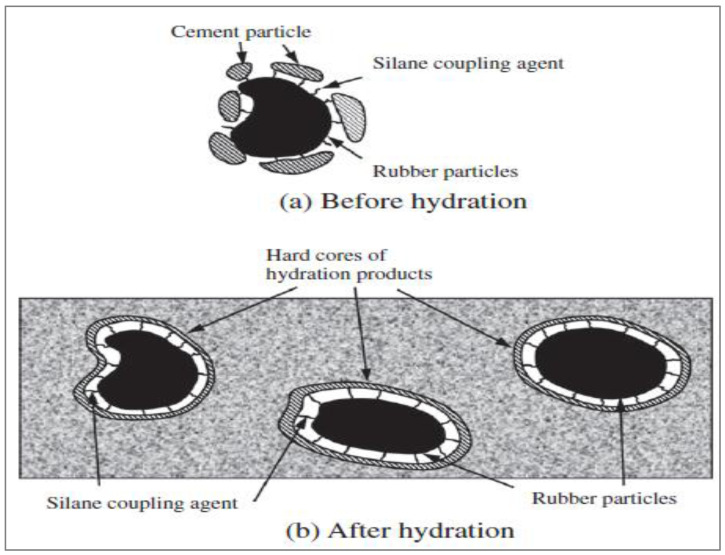
Mechanism of Cement Coating [105]: Used as per Elsevier permissions.

The hard shell that formed around rubber particles because of cement hydration, as shown in Figure 18, enhanced the stiffness compatibility between rubber and cement paste, which was the cause of the strength gain. There were some visible patches without a cement coat. It can be noted that 30% of cement-coated rubber particles looked to be darker as compared to 60%. This clarified why the 60% cement coating improved the compressive capacity of rubberized concrete more than the 30 weight % coating. According to research, rubberized cement’s resistance might be strengthened by using tiny rubber particles that were only rinsed in water, improving the cement’s strength by 16%. Additionally, a technique of reformatory water soaking has been found to control rubber’s hydrophilicity. Prior to mixing, the rubber is submerged in water for 24 h. Silica fumes (SF), mortar, cement paste (LP), and limestone powder are examples of pre-cementitious materials that are often employed. By covering the rubber with cementitious materials, the automatic properties of various rubberized cement may be effectively enhanced. A concrete matrix, cement coating, or rubber particles treated with silane coupling agents (SCAs) are more tightly coupled than untreated ones, according to the splitting tensile strength test. The coated rubber retained cement hydration products on its outside, while the uncoated rubber was left exposed. Typically, rubber processing entails air-seasoning the outside and coating the rubber with cementitious materials [106]. 

Rubber’s elastic modulus and interfacial transition zone (ITZ) adhesion were both enhanced by the procedure of coating it with cementitious ingredients. As a result, it was very successful in enhancing the strength and durability characteristics of rubberized concrete. Additionally, the basic materials employed in this process have a variety of uses. They thus hold promise for industrial and large manufacture of rubberized cement for structural purposes [100]. Ordinary rubberized concrete (ORC) gets a coating treatment to increase bending resistance.

## 5. Durability 

### 5.1. Permeability and Water Absorption 

**Figure 19:**
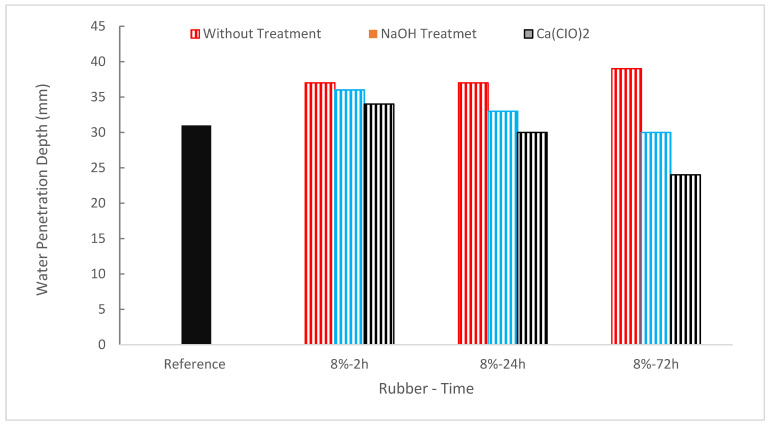
Water Penetration Depth: Data Source [39].

Figure 19 shows the water permeability of concrete with the substitution of rubber as aggregate. It can be noted that the permeability of concrete increased as the substitution ratio of rubber increased. However, the permeability of Ca(ClO)_2_-treated concrete is comparable to reference concrete. In concrete mixes, replacing rubber increases the depth of water permeability. In comparison to the second combination, the first mixture exhibits a greater rise in water permeability depth. Mixtures with substitutions of 5% and 7.5% rubber are categorized as having low permeability, while mixtures with replacements of 10% tire rubber are categorized as having medium permeability. In concrete mixes, replacing rubber improved water penetrability depth and enhanced water absorption when coarse aggregate was replaced but decreased water absorption when cement was replaced [67].

The results indicate that the Ca(ClO)_2_ treatment of rubber aggregates is the most successful method for lowering the permeability of concrete mixes. Additionally, the highest outcomes were seen during a 72 h therapy period. While the aggregates’ permeability was likewise decreased after being treated with NaOH, the improvement was noticeably less than after being treated with Ca(ClO)_2_. This decrease is also due to better rubber aggregate/paste bonding, which reduces the ITZ’s porosity [39]. For the samples containing waste rubber, a greater penetration depth was seen. This can be due to a decreased binding between rubber aggregate and cement paste and a greater water-cement ratio [67]. Additionally, the permeability of concrete has a considerable impact on its longevity. The water absorption test gauges the concrete’s capacity to transfer fluids [107]. The results of the tests showed that the existence of rubber tends to prevent water transmission and minimize water absorption, providing superior defense against corrosion for steel reinforcement [25].

The initial combination samples that were examined for water absorption looked to have fractured during oven drying, which led to noticeably higher values. The weaker link between the cement pastes and the bigger rubber particles (as opposed to the powder rubber in the second batch) may be the cause of this breaking. This rise is brought about by the substitution of rubber for sand, which has various forms and structures and develops some porosity, enhancing water absorption. Conversely, improving the quantity of fiber in concrete decreases water absorption [33].

The authors draw the conclusion that the water absorption is higher than it was for the control combination. This is because the connection between the cement pastes and big rubber particles has decreased. In contrast, when the proportion of replacement is raised, the water absorption of the second combination containing powdered tire rubber decreases. It seems that filling cavities with powdered rubber has decreased the porosity of the concrete, which has decreased water absorption in this combination [67]. By preventing water from spreading, rubber minimizes water absorption in concrete and helps to better protect the steel reinforcement against water [25].

### 5.2. Chloride Ion Penetration 

One significant unrecognized risk to the safety of buildings is the durability of concrete in the sea environment. The impact of dry-wet alternation substantially speeds up the diffusion of corrosive ions, and the tidal range region develops the maximum severe area for concrete structure corrosion. Among them, chloride ion diffusion is one of the primary causes of the durability of concrete [108].

**Figure 20:**
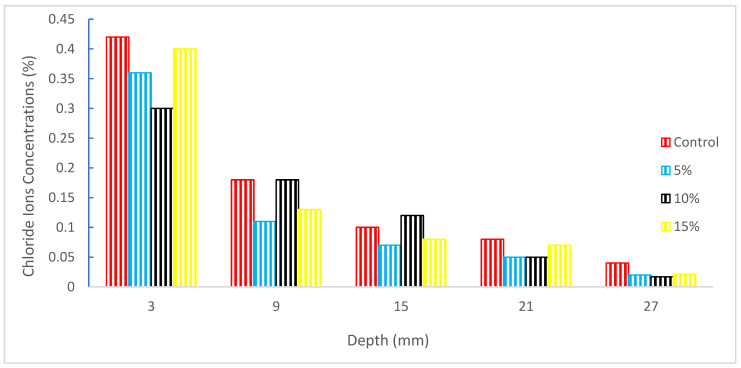
Chloride ion penetration: data source [109].

Figure 20 shows that adding rubber to concrete significantly lowers the absorption of free chloride ions in various depths of concrete and that the capability of concrete to resist chloride ion erosion is in the order of RC-2 group > RC-1 group > RC-3 group > OC group, indicating that adding the right quantity of rubber to concrete can enhanced concrete’s resistance to chloride ion destruction and decrease the interruption of chloride ions. It is recommended that the rubber percentage be 10% if only to lower the free chloride ion absorption at various depths of the concrete [109].

According to Oikonomou and Mavridou [110], as the quantity of rubber in mortar grew, the chloride ion diffusion dropped. When compared to the reference mix, there was a decrease of 14.22% in the 2.5% rubber mix and a reduction of 35.85% in the 15% rubber mix. Comparing concrete with 12.5% tire rubber to control mix concrete, the mixture with the bitumen emulsion showed a decrease in chloride ion penetration of up to 55.89%. On rubberized concrete, Bravo and Brito [111] tested for chlorine migration. For 5–15 replacement with tire rubber, an increase in chloride diffusion coefficient was seen. The chloride diffusion coefficient rises as rubber aggregate size increases. Concrete with tire aggregates ground mechanically provided greater resistance to chloride penetration than concrete with tire aggregates ground cryogenically. Chloride penetration was reduced when the curing time was extended.

Rubber may prevent concrete fractures from forming and can lower the peak value of steel corrosion, according to research by Jian Liang et al. [112]. Han Zhu [113] investigated the resistance of rubber concrete to chloride ion penetration under various conservational temperatures and discovered that rubber can decrease reinforcement corrosion and that the durability of rubber concrete is altered with various environmental temperatures. The chloride ion transport and erosion process of rubber concrete were thoroughly investigated and estimated by Han Qinghua et al. [114]. Using both macroscopic and microscopic simulations. The findings demonstrate that rubber has the capacity to significantly lower the chloride ion diffusion coefficient and increase the robustness of concrete structures.

According to research [115], replacing some of the coarse and fine aggregates with crumb rubber and rubber chips, respectively, gradually increased the diffusion of chloride ions. When silica fume was put into the concrete mix, it was discovered that the penetration was reduced. The increased resistance was attributed to the silica fume’s ability to fill spaces in cement pastes and transition zones between aggregate and paste. The addition of silica fume causes a decrease in the permeability of chloride ions. The cause was linked to the mortar’s decreased calcium hydroxide content [116].

## 6. Elevated Temperature

**Figure 21:**
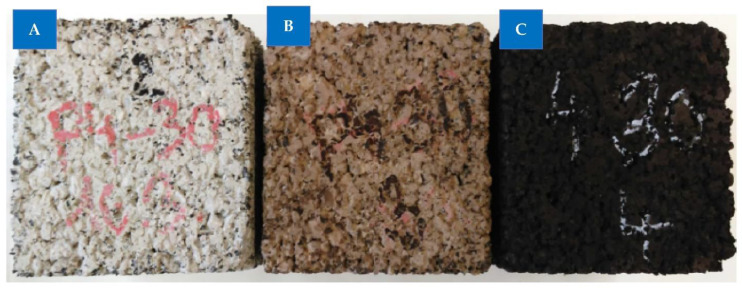
Effect of elevated temperature on rubberized concrete: (**A**) unheated, (**B**) 300 °C, and (**C**) 400 °C [45]: used as per Elsevier permission.

It was nearly impossible to see any difference when samples of rubberized concrete were exposed to temperatures of 150 °C and 200 °C. Figure 21 compares samples of material containing 30% rubber aggregate after being heated to 300 and 400 degrees Fahrenheit to a sample that was not heated. The reference white-grayish color of the sample shifted to a light brown and black hue. It resulted from the acceleration of rubber particle dissolution at higher temperatures, which began at about 300 °C. The subsequent burning of the rubber produced a black color.

All samples had an increase in mass loss as the heating temperature rose. The early mass loss was caused by the evaporation of capillary and gel water from the cement matrix, followed by the escape of absorbed and interlayer water, as demonstrated by the thermogravimetric measurement of rubber particles, which displayed that rubber disintegration begins quickly on the attainment of a temperature of 300 °C. The delivery of chemically bonded water, which is a component of cement hydration results and is particularly resistant to evaporation, may be responsible for the mass loss at higher temperatures [117]. The CSH phase’s dehydration process occurred at temperatures as high as 300 °C [118]. Ettringite and mono sulfate phases are also dehydrated between 110 and 156 °C, according to Liu et al. [119]. According to other scientists, the mass loss of all samples was comparable up to 300 [62]. At 400 °C, the mass loss for samples including rubber increased significantly, but the control sample only had a little rise of around 2.6%. The reference sample revealed the same mass loss result (2.6%) for 400 °C as Medine et al. [120]. It is clear that at 400 °C, the mass loss improved as the concrete’s rubber-based aggregate content rose.

When related to the strength characteristics found for materials held at laboratory temperature, a study [45] found that both the compressive and tensile capacity increased at a temperature of 150 °C. Gupta et al. [117] showed a similar rise in compressive capacity up to 150 °C and suggested that this rise may be related to a decrease in calcium hydroxide and un-hydrated area fraction, which is advantageous for the development of the compact concrete microstructure. On the other hand, Guelmine et al. [62] found that the damage factor rose with an increase in the used raised temperature of recycled rubber mortar, affecting both the CS and FS. For samples subjected to temperatures of 200 °C, 300 °C, and 400 °C, respectively, this concrete performance was seen. Damage factors between control concrete and rubberized concrete with varying amounts of rubber were mostly convergent up to 300 °C. A significant increase in the damage factor was seen during exposure to 400 °C, and this rise varied depending on the amount of rubber present. Typically, a steep slope of the damage factor function was seen for mixtures with significant rubber content. This conclusion is consistent with findings published, for instance, by Thomas and Gupta [121]. For control concrete, the major source of the damage processes is the temperature gradient that is applied to the samples, which results in water evaporation and the breakdown of the CSH, ettringite, and mono sulfate phases [122]. The burning of the rubber-based aggregate, which produced gaps and hence amplified its permeability, was specifically to blame for the extra damage to rubberized concrete at 400 °C.

## 7. Microstructure Analysis 

**Figure 22:**
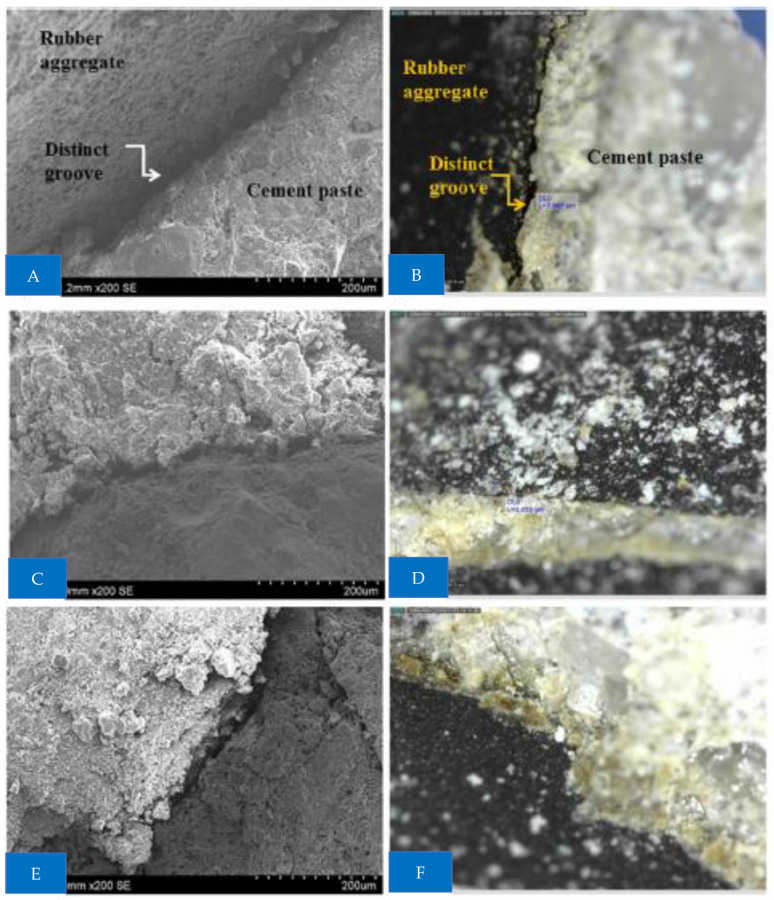
SEM results: (**A**,**B**) water, (**C**,**D**) NaOH treated, and (**E**,**F**) Ca(CIO_2_) treated [39].

Scan electronic microscopy (SEM) and digital microscopic pictures of concretes with rubber aggregates treated for 72 h with water, NaOH, and Ca(ClO)_2_ were collected for microstructural investigations are shown in Figure 22. Interfacial regions were photographed. When rubber aggregates were treated with water, distinct grooves with a gap width of around 7 mm were seen at the interfacial transition zone (ITZ). After being exposed to NaOH for 72 h, the rubber aggregate particles narrowed to 2.4–3 mm. There are no gaps at the interface after a 72 h Ca(ClO)_2_ treatment. This is explained by stronger aggregate/matrix bonding brought on by the rough tire surface and decreased porosity brought on by potential Friedel’s salt production. This is consistent with the strength data, which demonstrated that treating aggregates with Ca(ClO)_2_ for 72 h almost totally neutralized the strength decline shown for rubber particles that were not treated.

According to Pelisser et al. [53], treatment with NaOH and the addition of silica fume (SF) may produce an ITZ that is denser than concrete with untreated rubber. There is a noticeable reduction in the porosity of the rubber–cement matrix contact. The compressive strength of ORC after 28 days is just 14% lower than that of the reference concrete without rubber. The rubberized concrete mixture also includes natural zeolite to help rubber react with the NaOH solution treatment [31]. The rubber’s surface may be modified using NaOH solution to strengthen the connection between it and the cement. The mechanical qualities of rubberized concrete gradually enhance as a result of the synergistic interaction between natural zeolite and NaOH treatment.

When rubber is treated with a NaOH solution, the ITZ porosity of rubberized concrete is reduced, and the rubber–cement adhesion is increased, which lowers the mixture’s resistivity [123]. In order to improve adhesion between the two materials, SCA may be used as an aggregate or as an adhesion promoter at the interface between the rubber and cement matrix. It works to join the rubber and cement matrix chemically and physically into a tightly bound structure by acting on the organic/inorganic interface [102].

## 8. Applications of Rubberized Concrete

When compared to traditional concrete, rubberized concrete is more resilient to pressure, impact, and temperature. It is also cheaper and more cost-effective. It has been noted that the compressive and tensile strengths of rubber-modified concrete (RMC) are quite low. However, they have increased acid resistance, minimal shrinkage, strong impact resistance, suitable water resistance with low absorption, and great sound and thermal insulation. In comparison to a standard concrete mix, studies reveal that CRC (crumb rubber concrete) specimens stayed intact after failure (did not shatter). For construction that has to have strong impact resistance qualities, this behavior could be advantageous. Concrete samples aggregated with thick rubber were notably noticeable to have rubberized concrete with improved impact resistance.

Additionally, the special properties of rubberized concrete will find new applications in building structures for use as earthquake shock-wave absorbers, sound barriers, and highway construction as shock absorbers. It lessens plastic shrinkage, cracking, and concrete’s susceptibility to disastrous collapse.

Presently, precast sidewalk panels, non-load-bearing building walls, and precast roofs for green buildings all employ concrete that has had scrap tires added to it [124]. It is often used for construction-related tasks such as constructing ramps that are skid-resistant and constructing walkways and courts for enjoyment. These concretes are anticipated to be applied in architectural applications such as nailing concrete, where high strength is not required, wall panels that need low unit weight, construction elements, and impact-prone Jersey barriers, and railroads to secure rails to the ground, thanks to this new property [22].

Waste tire-modified concrete mixes might provide a feasible replacement to the standard weight concrete since rubberized concrete can also be utilized in non-load-bearing elements such as lightweight concrete walls, building facades, or other light architectural units [41]. Wherever cement-stabilized aggregate bases are required, especially below flexible pavements, rubberized mixes may be employed. The other practical uses may also be poured in bigger sheets than regular concrete and are ideal for usage in regions that often freeze and thaw.

## 9. Conclusions

The practice of rubber tires in concrete undoubtedly offers advantages, and the building sector as a whole cannot ignore this trend. For instance, rubber tires may be utilized as a substitute for natural aggregates. More than 100 modern and historical pieces of literature were evaluated to study the impact of rubber tires on the strength characteristics, freshness, and durability of rubberized concrete. The following were the key findings.

- Increase in rubber concentration. Rubberized concrete loses workability. However, it may be enhanced by adding admixtures such as plasticizers or other filler ingredients;
- The lower specific gravity and tendency to absorb air of rubber, rubberized concrete density reduces significantly when rubber content is increased. Rubberized concrete is hence advantageous for lightweight buildings;
- Concrete’s mechanical strength may generally be decreased by adding rubber, and this tendency becomes worse as rubber content rises. Due to the poor adherence of rubber with cement paste, a broad and porous weak interfacial transition zone (ITZ) was seen in rubberized concrete. The detrimental effects of rubber on the strength qualities of regular concrete may be lessened if the bond is strengthened at ITZ by any practical and affordable techniques. As a result, the construction industry would be able to employ rubberized concrete efficiently in a variety of concrete buildings;
- The decline in flexural capacity was lower than the decline in compressive capacity;
- The majority of studies feel that rubberized concrete with NaOH treatment has improved mechanical qualities. Other studies, however, asserted that the strength characteristics of rubberized concrete that has been treated with NaOH solution remain unchanged or even improve. The inconsistent findings might have been caused by varying rubber particle sizes, rubber suppliers, solution concentrations, and processing times;
- The silane coating agents (SCA) process transforms the rubber’s hydrophobic surface into a hydrophilic one and creates a chemical link between it and the cement matrix, enhancing the rubberized concrete’s mechanical characteristics and durability.

## 10. Recommendations

- Rubberized concrete performs badly at the moment. Pozzolanic filler additives could make it perform better. However, more detailed research is required before it may be used in a practical setting;
- The microstructure of rubberized concrete should be properly studied;
- Steel reinforcing bars’ corrosion behavior in rubberized concrete is recommended to be explored;
- Rubber surface treatment raises the price of utilizing rubber as a concrete aggregate. The cost of rubber surface modification should be investigated to evaluate its cost-effectiveness and identify the cheapest and most effective approach, which is crucial for more field applications;
- The thermal properties of rubberized concrete should be explored in more detail;
- The dry shrinkage and freeze–thaw action of rubberized concrete should be studied in detail.

## Figures and Tables

**Table 1:** Summary of slump flow.

Reference	Rubber Tire	Slump (mm)
[26]	0%, 25%, 50%, and 75%	180, 220, 215, and 215
[31]	0%, 5%, 10%, and 15%	80, 75, 64, and 55
[32]	0%, 5%, 10%, 15%, 20%, and 25%	0, 0, 7, 20, 55, and 87
[30]	0%, 10%, 20%, 30%, 40%, 50%, 60%, 70%, 80%, 90%, and 100%	215, 205, 200, 195, 180, 175, 150, 135, 135, 130, and 125
[33]	0%, 5%, 10%, and 15%	74.50, 74.00, 72.50, and 70.00
[34]	0%, 6%, 12%, 18%, 24%, 30%, 36%, 42%, 48%, 54%, and 60%	140, 138, 139, 130, 110, 70, 75, 22, 20, 10, and 0

**Table 2:** Summary of compressive strength.

Reference	Rubber Tire	Compression Strength (MPa)
[45]	0%, 10%, 20%, and 30%	61.5, 28, 11, and 3.5
[63]	0%, 25%, 50%, 75%, and 100%	31.9, 19.6, 13.8, 9.9, and 7.5
[70]	1%, 2%, and 5%	20, 15, and 12
[74]	0%, 5%, 10%, 15%, 20%, 25%, and 30%	54, 50, 45, 40, 35, 36, and 30
[75]	0%, 5%, 7.5%, 10%, 12.5%, 15%, 17.5%, and 20%	71.0, 70.5, 68.8, 66.5, 61.3, 54.8, 47.5, 37.3, and 30.3
[67]	0%, 5%, 7.5%, and 10%	32, 35, 30, and 25
[26]	0%, 25%, 50%, and 75%	45.80, 23.90, 20.87, and 17.42
[31]	0%, 5%, 10%, and 15%	27, 21, 17, and 12
[30]	0%, 10%, 20%, 30%, 40%, 50%, 60%, 70%, 80%, 90%, and 100%	41.5, 39.2, 36.4, 29.5, 22.8, 16.6, 13.3, 10.9, 9.20, 8.20, and 7.10
[35]	0%, 5%, 10%, 15%, and 20%	65, 60, 50, 40, and 35
[76]	0%, 5%, 10%, and 15%	55, 45, 35, and 25
[39]	0%, 8%, 10%, 20%, and 30%	38, 32, 27, 15, and 13
[33]	0%, 5%, 10%, and 15%	78.05, 68.12, 59.94, and 55.15
[77]	0%, 5%, 7.5%, and 10%	71, 68.8, 66.5, and 61.3
[78]	0%, 5%, 10%, 15%, 20%, 25%, 30%, and 35%	40, 44, 45, 38, 37, 37, 36, and 34
[79]	0%, 10%, 20%, and 30%	37.4, 40, 28.3, and 24.8
[80]	0%, 20%, 40%, 60%, 80%, and 100%	25.3, 18.9, 12.2, 8.0, 4.4, and 2.5
[81]	0%, 5%, 10%, 20%, and 30%	64, 46, 34, 14, and 10

**Table 3:** Summary of tensile strength (TS).

Reference	Rubber Tire	Tensile Strength (MPa)
[76]	0%, 5%, 10%, and 15%	4.2, 4.0, 3.5, and 3.0
[70]	1%, 2%, and 5%	2.7, 2.0, and 0.8
[74]	0%, 5%, 10%, 15%, 20%, 25%, and 30%	3.2, 2.7, 2.6, 2.5, 2.3, 2.2, and 2.1
[67]	0%, 5%, 7.5%, and 10%	3.0, 2.0, 1.6, and 1.4
[31]	0%, 5%, 10%, and 15%	3.02, 2.50, 2.33, and 2.04
[30]	0%, 10%, 20%, 30%, 40%, 50%, 60%, 70%, 80%, 90%, and 100%	4.38, 3.85, 3.67, 2.98, 2.51, 1.93, 1.52, 1.12, 0.98, 0.87, and 0.79
[35]	0%, 5%, 10%, 15%, and 20%	4.7, 4.5, 4.3, 4.0, and 3.7
[33]	0%, 5%, 10%, and 15%	4.90, 4.82, 4.63, and 4.20
[77]	0%, 5%, 7.5%, and 10%	3.0, 3.0, 1.5, and 1.4
[79]	0%, 10%, 20% and 30%	3.6, 2.3, 2.7, and 2.3
[80]	0%, 20%, 40%, 60%, 80%, and 100%	2.8, 1.8, 1.4, 0.9, 0.5, and 0.2
[81]	0%, 5%, 10%, 20%, and 30%	3.48, 3.68, 3.08, 1.83, and 1.70

**Table 4:** Summary of flexural strength (FS).

Reference	Rubber Tire	Flexure Strength (MPa)
[45]	0%, 10%, 20%, and 30%	6.8, 5.7, 3.1, and 1.5
[63]	0%, 25%, 50%, 75%, and 100%	3.8, 3.5, 3.1, 2.8, and 2.4
[70]	1%, 2%, and 5%	3.0, 3.0, and 4.2
[75]	0%, 5%, 7.5%, 10%, 12.5%, 15%, 17.5%, and 20%	7.2, 7.3, 6.9, 6.9, 6.6, 6.1, 5.7, 5.7, and 5.5
[67]	0%, 5%, 7.5%, and 10%	5.3, 5.2, 3.8, and 3.4
[26]	0%, 25%, 50%, and 75%	3.52, 2.93, 2.52, and 2.52
[31]	0%, 5%, 10%, and 15%	4.77, 5.97, 4.32, and 3.87
[32]	0%, 5%, 10%, 15%, 20%, and 25%	3.9, 3.8, 3.6, 3.3, 3.1, and 2.7
[30]	0%, 10%, 20%, 30%, 40%, 50%, 60%, 70%, 80%, 90%, and 100%	4.68, 4.44, 4.05, 3.57, 3.24, 2.79, 2.23, 1.81, 1.53, 1.08, and 0.87
[35]	0%, 5%, 10%, 15%, and 20%	7.0, 6.5, 6.0, 6.0, and 5.5
[76]	0%, 5%, 10%, and 15%	8.4, 8.0, 6.0, and 5.0
[33]	0%, 5%, 10%, and 15%	8.45, 8.03, 7.48, and 6.98
[77]	0%, 5%, 7.5%, and 10%	7.2, 6.9, 6.9, and 6.6
[78]	0%, 5%, 10%, 15%, 20%, 25%, 30%, and 35%	5.5, 6.0, 5.4, 5.1, 5.0, 4.5, 4.4, and 4.1
[80]	0%, 20%, 40%, 60%, 80%, and 100%	3.6, 2.5, 2.0, 1.3, 0.77, and 0.64
[81]	0%, 5%, 10%, 20%, and 30%	0.25, 0.32, 0.41, 0.25, and 0.19

## Data Availability

All the data are available in main text.
